# Effects of Carbapenem consumption on the prevalence of Acinetobacter infection in intensive care unit patients

**DOI:** 10.1186/1476-0711-13-7

**Published:** 2014-01-09

**Authors:** Aziz Ogutlu, Ertugrul Guclu, Oguz Karabay, Aylin Calica Utku, Nazan Tuna, Mehmet Yahyaoglu

**Affiliations:** 1 Sakarya University, Faculty of Medicine, Department of Infectious Diseases and Clinical Microbiology, Sakarya, Turkey; 2 Sakarya University, Health Science Institute, Sakarya, Turkey

**Keywords:** Carbapenem, *Acinetobacter* infection, Carbapenem consumption

## Abstract

**Background:**

The consumption of carbapenems has increased worldwide, together with the increase in resistant gram negative bacilli. Subsequently, the prevalence of carbapenem-resistant *Acinetobacter* infections has increased rapidly and become a significant problem particularly in intensive care unit patients. The aim of the present study was to evaluate the changes in the prevalence of *Acinetobacter* infection by restricting the consumption of carbapenems in intensive care unit patients.

**Methods:**

This study was conducted between May 1, 2011 and February 28, 2013. The amount of carbapenem consumption and the number of patients with multi-drug resistant *Acinetobacter baumannii* (MDRAB) isolates during the study period were retrospectively obtained from the records of the patients, who were hospitalized in the intensive care unit. The study period was divided into two periods named as: Carbapenem non-restricted period (CNRP) and carbapenem-restricted period (CRP). During CNRP, no restrictions were made on the use of carbapenems. During CRP, the use of carbapenems was not allowed if there was an alternative to carbapenems. Primary Endpoint: MDRAB infection after ICU admission. The definition of nosocomial infections related to *Acinetobacter spp*. was based on the criteria of the Center for Disease Control (CDC). The correlation between the amount of carbapenem consumption and the number of infections with MDRAB strains between the two periods were evaluated.

**Results:**

During the study period, a total of 1822 patients’ (1053 patients in CNRP and 769 patients in CRP) records were evaluated retrospectively. A total of 10.82 defined daily dose (DDD/100 ICU days) of anti-pseudomonal carbapenem were used in CNRP, and this figure decreased to 6.95 DDD/100 ICU days in CRP. In the 8-month CNRP, 42 (3.98%) MDRAB-related nosocomial infections were detected, and 14 (1.82%) infections were detected in CRP (p = 0.012).

**Conclusion:**

The prevalence of MDRAB strains isolated in the CNRP was 2.24-fold higher than the prevalence in the CRP. The prevalence of *Acinetobacter* infections can be reduced by taking strict isolation measures as well as by implementing good antibiotics usage policy.

## Background

Nosocomial infections (NI) caused by multidrug-resistant gram negative bacilli have become a significant problem in recent years [1]. Micro-organisms isolated from NI vary depending on the changes in antibiotic usage policy. *Acinetobacter spp.* has an important place among the causes of NI [2].

*Acinetobacter spp.* rarely causes infections in a normal host due to their low virulence. However, this agent is the most important cause of infections in intensive care units (ICUs). *Acinetobacter spp*. cannot be totally eliminated from intensive care units despite numerous established infection control measures. NIs caused by *Acinetobacter spp*. are associated with morbidity and mortality, and lead to increases in the length of hospital stay, as well as in treatment costs [3]. *Acinetobacter spp.* remains viable for a long period of time in a hospital setting, and can be isolated from soil, water, and food. The bacteria can colonize in healthy individuals and in hospitalized patients [4]. One of the most important factors that play a role in the spread of *Acinetobacter* spp. is the ability of bacteria to easily develop resistance to antibiotics. In the 1990s, most *Acinetobacter* spp. was sensitive to quinolones and carbapenems. However, hospital outbreaks caused by multiple-drug-resistant *Acinetobacter baumannii (MDRAB)* are reported in many countries in the last 10 years [5]. The infection with resistant strains was associated with higher mortality rates, longer hospital stays, and higher treatment costs [6].

In order to develop a common language for the resistance problem, bacteria resistant to ≥3 antibiotics are called multi-drug resistant (MDR) bacteria, and those resistant to all antibiotics are called pan-resistant bacteria [7]. The most important risk factor for the infections caused by MDR strains is the previous use of broad spectrum antibiotics [8]. Carbapenems are the most effective antibiotics in the treatment of infections caused by resistant strains of *Acinetobacter spp*[9]. However, carbapenem-resistant *Acinetobacter* strains are increasing worldwide [2]. Previous studies have reported infection and/or colonization of carbapenem-resistant *Acinetobacter baumannii* as the independent predictor of previous use of carbapenems [10-12]. Moreover, undesired affects in bacterial ecology occurring as the result of antibiotic therapy are called collateral damage (CD). CD implies antibiotic-resistant bacteria, multi-drug resistant bacteria, and infections and colonization by *Clostridium difficile*[13,14]. Antimicrobial agents used for treatment purposes cause CD by affecting not only the infectious agent but also microflora. CD is more frequently pronounced with the use of broad spectrum antibiotics in the hospital setting. The selection rate of the resistant bacteria varies depending on the type of antibiotics administered. Antibiotics most commonly associated with CD include third generation cephalosporins, fluoroquinolones, and carbapenems [14,15].

Can restriction of the use of carbapenems, which are effective against *Acinetobacter spp*, result in a change in the prevalence of MDRAB? There are limited studies addressing this question. The present study was aimed to evaluate changes in the prevalence of MDRAB-related infections with the restriction of the use of carbapenems.

## Methods

### Study design and data collection

The present study was conducted in a 550-bed tertiary care hospital in Marmara region, Sakarya (Turkey), retrospectively. We evaluated the medical records of patients who were hospitalized in the intensive care units of Sakarya University Training and Research Hospital (STRH) from May 1, 2011 to February 28, 2013. There were 24 beds in ICUs in the study periods. The bed capacity was the same in the both periods. The average of 1360 ICU patients were followed over the last four years in ICUs (1141, 1305, 1356, and 1403 respectively). Both surgical and internal patients who need intensive care were followed in the general ICU. Neurological patients were followed in the neurology ICU. Patient characteristics were similar in the periods. Reanimation specialist was followed the patients in the general ICU. Neurologist was followed the patients in the neurology ICU. Infectious diseases specialists were consulted ICUs every day. The amount of carbapenem consumption and data relevant to isolated MDRAB strains were obtained from the medical charts.

### Study periods

#### The study was conducted in two periods

Carbapenem non-restricted period (CNRP) and carbapenem-restricted period (CRP).

#### CNRP

Between May 1, 2011 and February 28, 2012. During this period, consulting physicians from the department of infectious diseases (CP) used carbapenem without any restriction in conditions they deemed appropriate. Carbapenem was not prescribed unless approved by the CP.

#### CRP

Between May 1, 2012 and February 28, 2013. The carbapenem usage was restricted during this period in the presence of an alternative therapy. The approval of the director of the department was sought for the use of carbapenem. Other CPs preferred alternative options other than carbapenem. During the carbapenem-restricted period, other options including piperacillin-tazobactam, cefoperazone-sulbactam, cefepime, tigecycline, and colistin were allowed. No restriction was allowed if the strain was sensitive only to carbapenems.

#### Antibiotic consumption data

Normally, all of the specialists could prescribe all antibiotics except 15 ones that required approvement by the infectious diseases specialist. MDRAB isolations were increased despite all of the infection control measurements. Moreover the Acinetobacter baumannii isolations were not susceptible to any drug except colistin. Some patients treatment were failed due to MDRAB infections. We searched the literatures, and then we decided to carbapenem restriction according to directives of infection control committee chairman. After the CNRP, we explained the carbapenem restriction and restriction requirement to the doctors. We did not do anything except carbapenem restriction.

#### Primary end-point

MDRAB infection occurring after admission to the intensive care unit.

#### MDRAB case definition

Patients from whom MDRAB was isolated and who were administered with carbapenem or other therapies for ≥ 72 hours during CNRP and CRP.

#### Inclusion criteria

Patients from whom MDR bacteria was isolated and who were diagnosed with systemic infection or pneumonia, and catheter, urinary tract, and wound site infection (the definition of MDR-related systemic infection, pneumonia, urinary tract, catheter, or wound site infection was made according to the criteria of the CDC [16]).

Patients falling under the scope of item one and who received carbapenem or other therapy for at least 72 hours.

#### Exclusion criteria

Sensitive strains that did not meet MDR criteria.

Patients with missing data.

Patients who were considered to be colonization.

Patients in whom carbapenems are contraindicated.

Pregnant women.

Patients below the age of 18 years.

#### Multi-Drug Resistance (MDR)

Acquired non-susceptibility to at least one agent in three or more antimicrobial categories (ampicillin/sulbactam, aztreonam, ceftazidime, ciprofloxacin, gentamicin, imipenem, piperacillin-tazobactam and trimethoprim/sulfamethoxazole) or resistance to >1 agent in ≥3 different groups of antibacterial including carbapenems [17].

#### Statistical analysis

Epi-info (CDC, Atlanta, USA) 6.0 software was used in the statistical analysis. P values <0.05 were considered significant.

The study was designed as a retrospective study. Therefore, approval was not obtained from the ethics committee.

## Results

**Figure 1:**
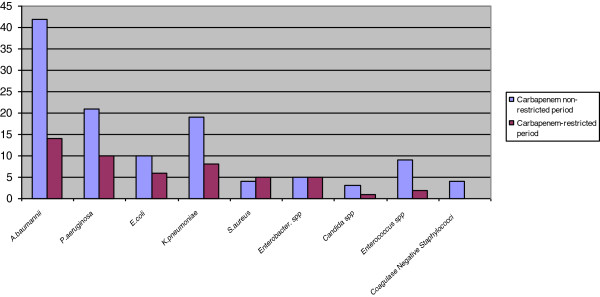
Distrubition of the isolated microorganisms in the study periods.

**Figure 2:**
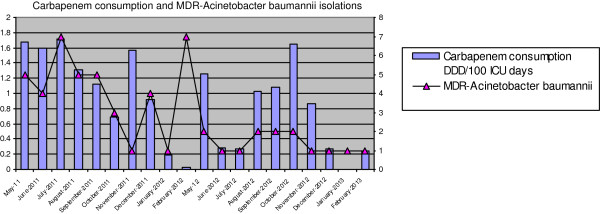
Carbapenem consumption and MDR-Acinetobacter baumannii isolations.

During the study period, a retrospective review of the records of 1822 patients (1053 patients (57.85) in CNRP and 769 (42.2%) patients in CRP) was evaluated. Carbapenem consumption was described as DDD/100 ICU days by months (Figure 1). A total of 10.82 defined daily dose (DDD/100 ICU days) of anti-pseudomonal carbapenem were used in CNRP, and this figure decreased to 6.95 DDD/100 ICU days in CRP. There was also an increase in the number of ertapenem (0.32 DDD/100 ICU days/0.76 DDD/100 ICU days) and amikacin (0.49 DDD/100ICU days/0.65 DDD/100 ICU days). During the study, MDRAB were isolated in total of 56 (3.1%) patients. MDRAB were isolated from 42 patients (3.98%) in CNRP, and from 14 patients (1.82%) in CRP (p = 0.012). The prevalence of MDRAB strains isolated in the CNRP was 2.24-fold higher than the prevalence in the CRP (Table 1). While *Pseudomonas aeruginosa* isolations were increased in the CRP, *Staphylococcus aureus* isolations were decreased (Figure 2).

**Table 1:** **The effect of carbapenem restriction on multi-drug resistant** ***Acinetobacter baumannii*** **isolation**

**Carbapenem restriction period**	**Number of patient n (%)**	**Number of patients with Acinetobacter isolated n (%)**	**Odds ratio (OR) (95% CI)**	**P value**
No	1053 (57,7)	42 (3,9)	2.24 (1.18-4.33)	0.012
Yes	769 (42,2)	14 (1,8)		
**Total**	**1822**	**56 (3,0)**		

Nosocomial infection density was 8.75 for 1000 patient days in CRP while nosocomial infection density was 19.70 in CNRP.

## Discussion

This study showed that the restriction of carbapenems in the intensive care units would cause a 2.24-fold reduction in the prevalence of MDRAB infections (p = 0.012). Accordingly, strictly restricting carbapenems in intensive care units may play a role in preventing outbreaks of MDRAB.

Anti-pseudomonal carbapenems are the choice of first line drugs in the treatment of infections by *Acinetobacter* spp. MDRAB strains are rapidly increasing due to the inappropriate use of carbapenems and other broad spectrum antibiotics. A 77% resistant rate has been reported in *Acinetobacter* strains isolated from ICU patients in 2012, in Turkey [18]. Therefore, carbapenems may not be the first choice in the ICU in Turkey. Uncontrolled consumption is one of the most important factors that rendered carbapenems useless in the treatment of *Acinetobacter* infections [19]. While depleting antibiotic arsenal for use in infections caused by resistant bacteria, further strategies need to be implemented to maintain the effectiveness of current antibiotics for longer duration in clinical use.

The prevalence of resistant *Acinetobacter* strains are higher in countries such as Greece, Turkey, and Italy, where carbapenems are intensely used, and the prevalence is lower in countries such as Holland, and Scandinavian countries [20,21].

Anti-microbial agents cause CD by not only affecting the infectious agent, but also affecting the micro flora of the hospital. The selection rate of the resistant bacteria varies depending on the type of antibiotics employed. Compared to other broad spectrum antibiotics, carbapenems also increase the colonization of MDR-*Acinetobacter,* MDR-*Pseudomonas aeruginosa,* carbapenemase-positive *Klebsiella* and *C.difficile* through CD [15,22]. In the present study, the isolation of a higher number of MDRAB in the CNRP is thought to be associated with CD. The antibiotics most commonly associated with CD include third generation cephalosporins, fluoroquinolones, and carbapenems [14,15]. In the present study, we think that lower prevalence of *Acinetobacter* infections in the CRP was due to the change in normal micro flora in the hospital.

In the literature, the studies suggest previous carbapenem consumption as a risk factor for MDR-*Acinetobacter* infections [23-25]. Our study results showed that all the microorganism isolations were decreased except *Staphylococcus aureus* in the CRP. A positive correlation was observed with anti-pseudomonal carbapenem (imipenem and meropenem) consumption and the development of resistance in *Acinetobacter* and *Pseudomonas* in a study which made in India [3]. MDRAB infections were shown to increase 16 times by using antipseudomonal carbapenems in a study conducted in Taiwan (19). Moreover, this increased resistance was not shown in any antimicrobial group except carbapenem. In this study, the importance of restriction in the use of antipseudomonal carbapenems was emphasized for the decreasing and controling of the MDR-*Acinetobacter* infection [19]. This situation may be due to selection of MDR-*Acinetobacter* which could be become resistant easly with broad-spectrum antibiotics such as carbapenems. We also think the requirements of rational antibiotic use policies for reduce the spread of MDR-*Acinetobacter spp* .

One study showed a significant reduction in the isolation of MDR-*Acinetobacter* strains in association with carbapenem restriction [26]. However, antibiotic stewardship programs alone are not sufficient to prevent *Acinetobacter* infections. Hand hygiene and other isolation measures are as important as antibiotic management.

Before any conclusion we should declare limitations of our study. This study was done only with a limited number of cases in a single center. If the number of cases were much more, study could has been more power. If we could be watched the genotypic changes in the bacterial resistance between the study periods, our results could have been more effective.

In conclusion, the strict restriction of carbapenem use is an important strategy to reduce the prevalence of MDR-*Acinetobacter* strains. However, all precautions (i.e. barrier measures, hand washing, sterilization, and disinfection) should be taken in intensive care units with the goal of achieving a "0%" infection rate.

## Competing interests

The authors declare that they have no competing interests.

## Authors’ contributions

AO and OK designed the study, prepared the literature, analyzed and interpreted the data, and wrote the manuscript; EG, ACU, NT and MY collected the data about carbapenem consumption and MDRAB isolates. All authors read and approved the final manuscript.
